# COVID-19 knowledge, attitude, and practice of United Arab Emirates heath providers at the start of the pandemic 2020

**DOI:** 10.1186/s13690-022-01015-w

**Published:** 2023-02-13

**Authors:** Aljazia Khalfan Alghfeli, Amal Abdul Rahim Al Zarooni, Hamda Musabbah Alremeithi, Roqayah Abdulla Almadhaani, Latifa Baynouna Alketbi

**Affiliations:** Family Medicine, Ambulatory Healthcare Services, Abu Dhabi Health Services Company, Abu Dhabi, United Arab Emirates

**Keywords:** COVID-19, Healthcare providers, Healthcare workers, Healthcare professionals, Coronavirus, Knowledge, Attitude, Practice

## Abstract

**Background:**

Early in 2020, healthcare providers faced the novel virus COVID-19 that had unprecedented risk to them and the community they serve. With COVID-19 high infectivity rate and considerable morbidity and mortality, healthcare providers ' precautionary practices to protect themselves, colleagues, and patients were determinantal to provide safe health services. This study aims to assess the knowledge, attitude, and practice of healthcare providers in the United Arab Emirates toward COVID-19 and to examine its determinants.

**Method:**

This cross-sectional study was conducted to assess COVID-19 knowledge, attitudes, and practice (KAP) of healthcare providers in the Emirates of Abu Dhabi, the United Arab Emirates, from April to July 2020, using an online anonymous self-administered questionnaire. A convenient sampling method was used to distribute the online survey link through the organization's email network admin list and smartphone messaging. Descriptive statistics, t-tests, and multivariant linear regression were used.

**Results:**

A total of 2371 healthcare providers responded to the survey. A total of 1091 worked in inpatient hospitals, 494 in primary health care, and 388 in emergency and ICU care. The overall performance score for all healthcare providers was as follows: 49.1%, poor score; 41.8%, intermediate score; and 9.2%, good score with a mean result of 17.14. Factors leading to better overall performance scores were years of experience, pediatricians’ specialty, and specialist occupation. A total of 55.7% received good direct knowledge from all healthcare providers. In practice, 48% had good practices toward COVID-19. The overall attitude mean was 2.8, with a maximum score of 7, indicating a positive attitude toward COVID-19.

**Conclusions:**

Although this study describes a dynamic learning status and could reflect the early pandemic situation in Abu Dhabi health care, it does provide a method to assess the precursor of the critical outcome. It is recommended to follow this study with an assessment of the training program targeting all healthcare providers to ensure a better response to emerging infections.

**Supplementary Information:**

The online version contains supplementary material available at 10.1186/s13690-022-01015-w.

## Background

The emergence of COVID-19 infection created a global health burden and public health crisis in many countries which has had a rapid surge in the number of cases and deaths [1–3]. COVID-19 was declared a global pandemic on March 11, 2020, by the World Health Organization (WHO) [4]. Covid-19 is responsible for mild to severe respiratory infections. Still, its very high transmission rate and the resulting high numbers of deaths have affected healthcare systems worldwide and, in many, went beyond their capacity. The pandemic has impacted, in addition to a great extent, their economy and all social life [5].

In the UAE, the government focused on supporting all healthcare facilities with all needed resources and staffing, including preventive measures and policies to control COVID-19 transmission among healthcare providers. This included implementing known effective measures such as workplace precautions such as frequent hand washing, wearing masks, and social distancing or vaccination [6]. Healthcare providers' efforts and practice are essential to control any possible transmission or further disease outbreaks caused by COVID-19 [7]. They have a higher risk of exposure and can acquire COVID-19 if personal protective measures were not used appropriately or were not practicing good precautionary procedures [8]. They are the frontline in facing the COVID-19 pandemic as they continue to care for patients despite the risk of personal infection and fear of transmission to them or their family members [7].

Poor knowledge about communicable disease and infection control practices places healthcare providers at increased risk. In 2002, during the SARS outbreak, approximately one-fifth of all cases were from healthcare providers [9]. With the importance of adequate knowledge and attitude in preventing infection, many studies demonstrated a gap in knowledge and inaccurate perceptions of COVID-19 risk among Healthcare providers [10, 11]. Health authorities in the United Arab Emirates (UAE) have released national COVID-19 guidelines, educational material, and online educational sessions for healthcare providers. Free access to online medical library resources was provided to all healthcare workers with access to guidelines, policies, procedures, and recommendations about COVID-19. Risk assessment and frequent periodic COVID-19 screening tests were performed for all healthcare providers in the United Arab Emirates [12]. In addition, mental health counseling, psychologist support, and helpline support were provided to the healthcare team in the United Arab Emirates [12].

This study aimed to assess the knowledge, attitude, and practice of healthcare providers in the United Arab Emirates toward COVID-19. In addition, to examining factors influencing their adherence to precautionary practice.

## Methods

### Study design, setting, and population

A cross-sectional study was conducted in the United Arab Emirates among healthcare providers from primary healthcare centers and hospitals in the private and public sectors. The survey was sent by internal organization email list and smartphone messaging to all Abu Dhabi Health Services (SEHA) staff. Participants were eligible for the study if adult healthcare providers who sign the online consent. The study was conducted from April to July 2020. The sample size was determined using a margin of error of 5% and a confidence interval (CI) of 95%. The required sample size was 383. Due to the distribution of the survey as online sampling, it was a convenient sample. Since this was conducted during the early stage of the COVID-19 pandemic in April 2020, and the tool design was based on a literature review and expert opinions. The tool utilized a mixed method of assessment that included real-life scenarios and direct knowledge questions. There was no previous validated tool in this area.

Data were collected using an anonymous online self-administered questionnaire developed in English. The questionnaire was designed to collect participants' demographic data (age, occupation, specialty, years of experience, city, and practice setting). The questionnaire was intended to collect information about healthcare providers’ knowledge, practice, and attitude about and toward COVID-19, information collected about basic knowledge on COVID-19, guidelines, mode of transmission, investigation, risk group, infection prevention, exposure risk assessment for healthcare workers, and possible actions following the risk assessment. Screening for depression and anxiety using PHQ9 and GAD 7 was performed. The questionnaire was developed by reviewing available questionnaires in the literature and the national SEHA, Centers for Disease Control and Prevention (CDC), and WHO guidelines. The questionnaire was then constructed based on expert opinions, available guidance from the local, SEHA, and UAE ministry of health, and international guidance from CDC and WHO. The question design is a case-based scenario. The questionnaire was piloted among 20 healthcare providers. Copy of questionnaires attaches to the supplementary material.

The scoring system was developed by assigning one score to each correct answer. The questionnaire included direct knowledge, practice, and attitude questions in base case seniors measure knowledge, practice, and attitude. The participants' overall performance was measured by considering the questionnaire as a quiz-like assessment of real-life situations. The total score of overall performance was 40 based on the 40 questions available in the survey. An overall performance score of more than 30 out of 40 indicates good performance, and an overall performance score of less than 20 indicates poor performance. Intermediate performance was assessed using a 20–30 performance score. There were 18 direct knowledge-related questions, seven attitude-related questions, and nine direct practice-related questions.

The knowledge section's total score ranges from 0 to 18, and a cut-off level of ≤ 8 was set as poor knowledge and ≥ 9 for good knowledge. The attitude section’s total score ranged from 0 to 7. The attitude score of 7 is the best, and 0 is the worst. The practice items total score ranged from 0 to 9, and a score of < 5 indicated poor practice toward precautionary measures for COVID-19.

The depression scoring system includes nine items. Each item had four responses. Each response had a specific score: not at all = 0, several days = 1, more than half a day = 2, nearly every day = 3. The total scores for depression items ranged from 0 to 27, 0 to 4 suggest minimal depression, 5 to 9 mild depression, 10 to 14 moderate depression, 15 to 19 moderately severe depression, and 20 to 27 severe depression.

The generalized anxiety disorder scoring system includes seven items. Each item has four responses, and each response has a specific score as follows: not at all = 0, several days = 1, more than half a day = 2, nearly every day = 3. Anxiety items’ total scores ranged from 0 to 21. A score of 0 to 4 suggests minimal anxiety, 5 to 9 mild anxiety, 10 to 14 moderate anxiety, and 15 to 21 severe anxiety.

### Statistical analysis

Descriptive statistics, such as mean, and standard deviation (SD), were computed for quantitative variables, and frequencies and percentages were calculated for categorical variables. All analyses were performed using SPSS 21 software program. Linear regression was used to test the association between a demographic variable and COVID-19 Knowledge, Attitude, and Practice of the Healthcare Providers. The significance value of ≤ 0.05 was set at a significant level.

### Ethics and confidentiality

All study participants were informed of the study. Online consent for participation was obtained before enrollment. This study was approved by the Abu Dhabi Health COVID-19 Research Ethics Committee DOH/CVDC/2020/242. The questionnaire was anonymous and did not include any identifiers or personal information of the participants. The confidentiality of the participants was maintained.

## Results

A total of 2371 SEHA healthcare providers responded to the survey, which is a good response rate as it represents 23% of SEHA Healthcare providers. Participants had a mean age of 39.94 years (SD 9.02) and were mostly female (1754, 74%). With regards to the site of work (1091, 46%) of them were working in inpatient hospitals, (494,20.8%) were working in primary health care, and (388, 16.4%) were working in emergency and ICU care settings. Most participants (1926, 81%) were from SEHA, and only (225, 9.5%) were from outside SEHA. Nurses constituted (1467, 61.9%) of the respondents and (445,18.8%) were physicians (consultants, specialists, residents), (330,13.9%) were technicians, and only (129, 5.4%) were pharmacists. Physicians who enrolled were from different specialties: family medicine (166, 7%); internal medicine, 8.6 (205,8.6%); obstetrics and gynecology (180, 7.6%); pediatrics (230, 9.7%); psychiatry (39, 1.6%); and surgeons, (208, 8.8%). Most participants were from Abu Dhabi and Al Ain (64%, 26.5%), respectively, and 8.1% were from the western region. The mean work experience was 14 years (SD 8.26). The demographic characteristics of the study population are summarized in Table 1.

**Table 1:** Characteristics of United Arab Emirates Heath providers at the start of the pandemic 2020

Age (years)	(Mean age /SD)	(39.94 years/ 9.02)
Sex % (N)	Female	74.0 (1754)
	Male	26.0 (617)
Occupation % (N)	Consultant	3.9 (93)
	Nurse	61.9 (1467)
	Pharmacist	5.4 (129)
	Resident	3.8 (90)
	Specialist	11.1 (262)
	Technician	13.9 (330)
Specialty % (N)	Family medicine	7.0 (166)
	Internal Medicine	8.6 (205)
	Obstetrics and Gynecology	7.6 (180)
	Other	56.6 (1343)
	Pediatrics	9.7 (230)
	Psychiatry	1.6 (39)
	Surgical specialties	8.8 (208)
Practice sitting %	Emergency /ICU	16.4
	Inpatient hospital-based	46
	Other	16.8
	Primary health care clinic	20.8
City %	Abu Dhabi	64.0
	Alain	26.5
	Other	1.3
	Western Region	8.1
Experience	Years of work experience (mean/SD)	(13.988 years / 8.26)

Regarding the healthcare providers' overall knowledge, attitude, and practice, more than half of them (55.7%) had good direct knowledge. In practice, 48% had good practices toward COVID-19. The overall attitude mean was also 2.8, from a maximum score of 7, indicating a positive attitude toward COVID-19.

The overall performance score based on participants' achievement from a total of possible 40 points if all were correct answers was mainly poor as half of the participants (49.1%) answered 20 or fewer correct answers. Another 41.8% scored between 20 and 30, and only 9.2% had a good performance score above 30 out of 40, meaning they would have chosen the right option for the cases given. The overall mean score was 17.14.

**Fig. 1:**
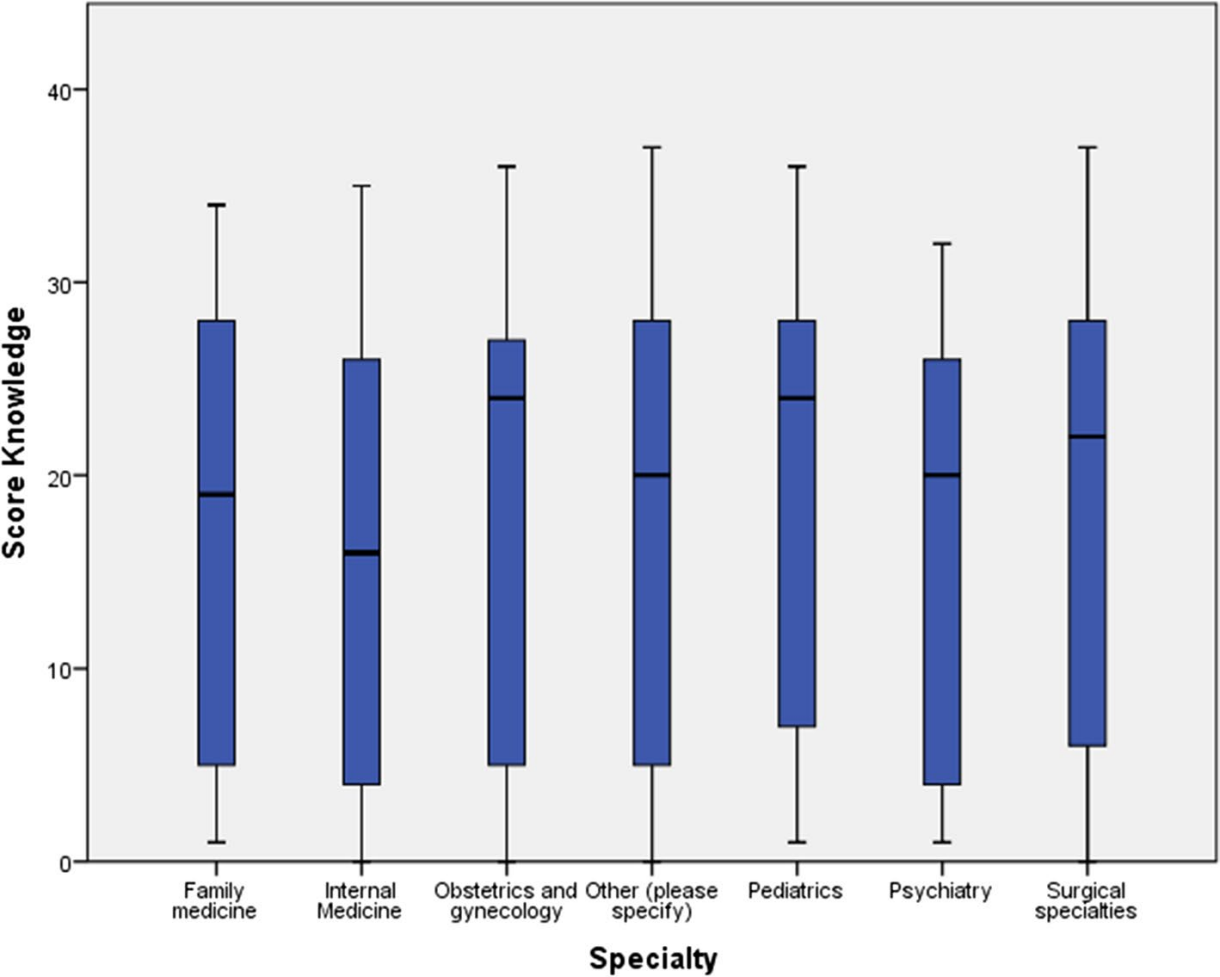
Overall performance about COVID-19 Knowledge score distribution among specialty of United Arab Emirates Heath providers at the start of the pandemic 2020

Figure 1 shows the overall performance scores among the specialties, with pediatricians having higher overall performance scores. Other key specialties in the pandemic were lagging, such as internal medicine and family medicine.

Regarding the direct knowledge questions, only half of the respondents indicated a correct answer about the mode of transmission of the COVID-19 virus, either by respiratory droplets or contact with contaminated surfaces. As well, only 32.3% believed that COVID-19 virus transmission could be via the oral route. The social distancing of 2 m or more was reported correctly by 43.3% of healthcare providers as the correct distance to prevent transmission of the COVID-19 virus. In comparison, 11.3% of all healthcare providers believed that 1 m or more was considered a safe social distance, and 0.1% reported that they did not know the exact social distance. More than half of healthcare providers reported the following as high-risk groups: age > 60 years (55.5%), smokers (50%), diabetes mellitus (53.6%), hypertension (50.3%), patients with chemotherapy (55.1%), and patients with asthma and COPD (55.1%). Pregnant women, 48.2%, were also reported as a high-risk group. Table 2.

**Table 2:** COVID-19-related knowledge, attitudes, and practice (KAP) of United Arab Emirates Heath providers at the start of the pandemic 2020

knowledge		occupation				
HCP response to a different aspect of knowledge:		Physician (n, %)	Nurse (n, %)	Pharmacist (n, %)	Technician (n, %)	Total % (n, %)
Mood of transmission	Respiratory droplets	257, 54.2%	808, 55.1%	64, 49.6%	186, 56.4%	1315, 55.5%
	Direct contact with contaminated surfaces	235, 49%	759, 51.7%	61, 47.3%	173, 52.4%	1228, 51.8%
	Oral route	147, 31.4%	449, 30.6%	43, 33.3%	128, 38.8%	767, 32.3%
Social distance	1 m (3 feet) or more	28, 6.4%	192, 13.1%	12, 9.3%	37, 11.2%	269, 11.3%
	2 m (6 feet) or more	232, 48.4%	630, 42.9%	54, 41.9%	157, 47.6%	1073, 45.3%
High-risk group	Age > 60	257, 54.4%	809, 55.1%	65, 50.4%	186, 56.4%	1317, 55.5%
	Smoker	227, 47.8%	727, 49.6%	59, 45.7%	171, 51.8%	1184, 49.9%
	Diabetic patient	253, 53.4%	775, 52.8%	64, 49.6%	178, 53.9%	1270, 53.6%
	Hypertensive patient	235, 50%	723, 49.3%	59, 45.7%	175, 53%	1192, 50.3%
	Patient receiving chemotherapy	260, 54.8%	804, 54.8%	63, 48.8%	180, 54.5%	1307, 55.1%
	Patient having Asthma or COPD	253, 53%	804, 54.8%	63, 48.8%	187, 57.7%	1307, 55.1%
	Pregnant female	219, 46.5%	712, 48.5%	54, 41.9%	157, 47.6%	1142, 48.2%
	Patient with GERD or peptic ulcer disease	51, 9.8%	323, 22%	20, 15.5%	80, 24.2%	474, 20%
	Thalassemia carrier	70, 14.3%	481, 32.8%	36, 27.9%	99, 30%	686, 28.9%
Sensitivity and specificity of COVID-19 testing in detecting the virus	The nasopharyngeal swab is recommended over the oropharyngeal swab	203, 42.8%	559, 38.1%	45, 34.9%	134, 40.6%	941, 39.7%
	The sensitivity of the NP swab is 70%	124, 27%	134, 9.1%	19, 14.7%	44, 13.3%	321, 13.5%
	The sensitivity of NP swab is 90%	72, 14,4%	263, 17.9%	20, 15.5%	67, 20.3%	422, 17.8%
	Specificity of NP swab is 70%	63, 13%	89, 6.1%	14, 10.9%	31, 9.4%	197, 8.3%
Wearing a surgical mask is an indication	Suspected COVID-19 infection patient	142, 29.7%	478, 32.6%	32, 24.8%	112, 33.9%	764, 32.2%
	Only patients with respiratory symptoms like cough or fever	77, 17%	315, 21.5%	22, 17.1%	84, 25.5%	498, 21%
	All community	189, 40.1%	640, 43.6%	48, 37.2%	152, 46.1%	1029, 43.4%
	Only medical staff and caregivers in close contact with patients	84, 18.6%	302, 20.6%	25, 19.4%	83, 25.2%	494, 20.8%%
Attitude						
The attitude of HCP if symptomatic without a history of contact with COVID-19 case or history of travel		55 11.5%	171 11.7%	19 15.5%	31 9.7%	276 11.7%
When practicing in a primary healthcare center	All employees are advised to check for any signs of illness and notify their supervisor if they become ill	242, 50.1%	753, 51.3%	56, 43.3%	171, 51.8%	1222, 51.5%
	Minimize interaction and interview time with patients	205, 43.3%	526, 35.9%	43, 33.3%	143, 43.3%	917, 38.7%
	Ensure to wear the face mask while in the clinic	245, 51.5%	784, 53.4%	65, 50.4%	188, 57%	1282, 54.1%
	Avoid being in an area like coffee room, or staff changing room	183, 37.7%	357, 24.3%	44, 34.1%	123, 37.3%	707, 29.8%
	Don't bring unnecessary stuff to the clinic like your personal lab top, notes, or textbooks	231, 48.3%	670, 45.7%	55, 42.6%	160, 48.5%	1116, 47.1%
	Keep your tools like a stethoscope in the clinic, and use disinfectant wipes to clean them frequently	233, 49%	744, 50.7%	56 43.4%	179 54.2%	1212, 51.1%
Practice						
The practice of HCP regarding action after having a negative swab in a symptomatic patient		318 65.5%	847 57.7%	55 64.3%	184 55.8%	1404 59.2%
The action of HCP after medium-risk exposure		147, 33.1%	556 37.9%	45 34.9%	123 37.3%	871, 36.7%
The action of HCP after brief low-risk exposure		170, 35.1%	513, 35%	36, 30.2%	114, 34.5%	833, 35.1%
The action of HCP after low-risk exposure while wearing full PEE		252, 53.7%	756, 51.7%	57, 44.2%	164, 49.7%	1229, 51.8%
The practice of HCP with contact of contact case	Wearing PEE	233, 49.8%	728, 49.6%	59, 45.7%	175, 53%	1195 50.4%
	Send the patient to the isolation room before assessment	183, 39.1%	483, 32.9%	39, 30.2%	106, 32.1%	811, 34.2%
	Reassure family and send home for self-monitoring and home quarantine	153, 31%	701, 47.8%	49, 38%	157, 47.6%	1060, 44.7%

The nasopharyngeal swab was reported by 39.7% to be recommended over the oropharyngeal swab for detecting the COVID-19 virus. Regarding the PCR test sensitivity and specificity, 17.8% of all healthcare providers believe that the sensitivity of the nasopharyngeal swab is 90%. In comparison, 13.5% think that the sensitivity is 70%, and 8.3% think that the specificity of the nasopharyngeal swab is 70% Table 2.

Wearing surgical masks was perceived by 32.2% of all healthcare providers as necessary for suspected COVID-19 infection patients, and 21% reported wearing surgical masks only with patients with respiratory symptoms such as fever and cough. While 20% reported that only medical staff and caregivers in close contact with patients should wear surgical masks, less than half of the healthcare providers (43.4%) reported that all communities should wear surgical masks Table 2.

The attitude of healthcare workers toward COVID-19 testing was assessed through the question, “what action they will take if they start to develop any symptoms like a dry cough in the absence of a history of contact with COVID-19 case or travel history. Nearly half of them (47.5%) reported that they would ‘‘do a COVID-19 PCR test’’ as an action, while 11.7% would continue to work without further action, and 6.3% would isolate themselves at home.

More than half of the HCWs practicing from the primary healthcare clinic reported that they would check for any signs of illness and notify their supervisors if they become ill, ensure wearing a face mask while in the clinic, and keep tools like the stethoscope in the clinic and use disinfectant wipes to clean it frequently; 51.1%,54.1%, and 51.1%). Similarly, 47.1% reported that they would not bring unnecessary staff to the clinic, and only 29.8% reported avoiding being in areas like coffee rooms and team changing rooms Table 2.

Of all healthcare workers, 59.9% correctly reported the action after having a negative swab in a symptomatic patient. Of those who gave correct answers, 57.7% were nurses, and 65.5% were physicians. The action of healthcare providers after medium-risk exposure assessment was correctly answered by 36.7% of all healthcare providers, of which 37.9% of nurses responded correctly, with 33.1% physicians and 37.3% technicians. Action after low-risk exposure assessment was correctly answered by 35.1% of healthcare providers and 51.8% of all healthcare providers answered correctly regarding low-risk exposure with full PPE Table 2.

In the case scenario of dealing with a patient who had contacted a positive case, 50% of all healthcare providers answered incorrectly that they would wear full PEE before dealing with such cases. Only 34.2% reported that they would direct the contact of a positive case to the isolation room. In addition, 44.7% of healthcare providers reported that they would reassure the family and send the case home with self-monitoring and home quarantine. Only 36.5% of all healthcare providers reported consistently practicing infection control precautions in primary healthcare, and only 1% reported never practicing infection control precautions Table 2.

Participants' assessment of the risk level of COVID-19 infection, high, intermediate, or low after a case exposure, was assessed through developed scenarios. Almost three-quarters of participants, 67.2%, correctly answered the question regarding high-risk assessment for a healthcare provider who was not wearing full PPE when exposed to a positive case of COVID-19, including not wearing a face mask. There was no difference in identifying the correct risk level between the different disciplines, as nurses, physicians, and technicians performed the same. Table 3. Using linear regression, consultants and residents significantly performed worse in high-risk assessment knowledge (B = -0.045, *P* = 0.030) and (B = -0.043, and *P* = 0.037, respectively). Technicians, on the other hand, did significantly better in high-risk assessment knowledge (B = -0.049, *P* = 0.016).

**Table 3:** Application of risk assessment knowledge about COVID-19 through cases of United Arab Emirates Heath providers at the start of the pandemic 2020

Risk Assessment	Occupation				
	Physician (n, %)	Nurse (n, %)	Pharmacist (n, %)	Technician (n, %)	Total % (n, %)
High-risk assessment of HCP exposure	275, 59.4%	989, 67.4%	88, 68.2%	241, 73%	1593, 67.2%
Medium-risk assessment of HCP exposure	123 25.9%	438, 29.9%	47 36.4%	103, 31.2%	711, 30%
Low-risk assessment of HCP exposure	40, 9.6%	87, 5.9%	8, 6.2%	19, 5.8%	154, 6.5%

Regarding healthcare providers’ exposure, while performing tooth extraction in a case of COVID-19, while wearing surgical masks and hand gloves with a medium-risk total exposure time of 20 min, 30% got the correct answer indicating medium risk Table 3 . Knowledge of medium-risk exposure assessment of healthcare providers was significantly and positively associated with the surgical specialty group (B = 0.48, *P*-value 0.019). Moreover, medium-risk assessment knowledge was significantly and negatively associated with age (B = -0.054, *P* = 0.009).

For the question on low-risk exposure assessment, most healthcare providers overestimated the risk of high-risk exposure (52%). The only significant performance of the study groups was that consultants did significantly better in identifying lower-risk situations (B = 0.079, *P*-value < 0.001).

Regarding the influence of different factors on knowledge, attitude, and practice, Table 4 shows that older age was significantly associated with better knowledge. Healthcare providers aged 41 to 50 had the best knowledge (59.8%) (*p* = 0.042). A higher mean for attitude score was noted in the same age group (41–50), but it was not significant (*p* = 0.054). On the other hand, higher knowledge (62.4%) and practice (51.5%) scores and attitude mean (3.48) were noted in the group with more years of experience (more than 30 years) as healthcare providers, but this as well was not significant (*p* = 0.055, *p* = 0.324, *p* = 0.375).

**Table 4:** Difference in health care provider’s knowledge, attitudes, and practice (KAP) about COVID-19 by demographics of United Arab Emirates Heath providers at the start of the pandemic 2020

Characteristics	Knowledge*			Attitude**			Practice***		
	Poor knowledge	Good knowledge	*P*-value	Mean	SD	*P*-value	Poor Practice	Good Practice	*P*-value
	(%)	(%)					(%)	(%)	
Gender			0.144						0.108
male	46.8	53.2		2.85	2.68		54.8	45.2	
female	43.4	56.6		2.80	2.77		51	49	
Age group			0.042			0.054			0.177
less than 30	48.1	51.9		2.6	2.68		56	44	
30–40	46.3	53.7		2.7	2.67		52.4	47.6	
41–50	40.2	59.8		3.03	2.69		48.7	51.3	
more than 50	42.5	57.5		3	2.79		53	47	
Years of experience			0.055			0.324			0.375
less than 5	44.1	55.9		2.8	2.7		55	45	
6–10	48.6	51.4		2.6	2.7		54.1	45.9	
11–20	43.8	56.2		2.8	2.66		50.1	49.9	
21–30	40	60		3.08	2.7		51.6	48.4	
more than 30	37.6	62.4		3.48	2.86		48.5	51.5	
Occupation			0.056			0.00			0.015
consultant	44.1	55.9		3.03	2.86		55.9	44.1	
Nurse	44.6	55.4		2.73	2.63		51.1	48.9	
pharmacist	49.6	50.4		2.62	2.77		60.5	39.5	
Resident	55.6	44.4		2.23	2.76		65.6	34.4	
Specialist	37.8	62.2		3.4	2.81		46.9	53.1	
Technician	43.3	56.7		3.01	2.76		52.1	47.9	
Specialty			0.018			0.014			0.039
Family medicine	47	53		2.75	2.73		52.4	47.6	
Internal medicine	48.8	51.2		2.37	2.53		58	42	
Obstetrics and gynecology	40.6	59.4		3.05	2.6		46.7	53.3	
Pediatric	35.2	64.8		3.37	2.67		46.1	53.9	
Psychiatry	43.6	56.4		2.7	2.59		69.2	30.8	
Surgery	38.9	61.1		3.05	2.69		50.5	49.5	
others	46.2	53.8		2.77	2.72		52.2	47.5	

^*^Knowledge section total score ranges from 0–18 and the cut-off level of < than or equal of 8 was set for poor knowledge and > or equal of 9 for good knowledge

^**^ Attitude section total score ranges from 0–7

^***^Practice items total score ranged as 0–9, and a score of < 5 indicates poor practice toward precautionary measures of COVID-19

For occupation, specialists had the highest scores in knowledge (62.2%), practice (53.1%), and attitude mean (3.4), with a significant P-value for practice (0.015). On the other hand, medical residents from all specialties had the lowest score, indicating poor knowledge (55.6%) and poor practice (65.6%). While Pediatrics had a significantly higher knowledge score (64.8%), P-value of (0.018), higher practice score (53.9%), *P*-value (0.039), and the highest means for the positive attitude of all specialties (3.37) Table 4.

Anxiety and depression scores were measured in a total of 1268 participants of healthcare providers. More than half of the participants reported anxiety symptoms (51.5%). Depression symptoms were revealed in 38.3% of participating providers. The detailed result of the mental screening was reported separately [13]. Depression was negatively associated with the overall performance scores, which means that those with good knowledge, practice, and positive attitudes had lower rates of depression (*P*-value 0.00).

With regards to the overall performance, using multivariate linear regression on all factors studied (gender, age, years of experience, occupation, specialty), only years of experience, being a pediatrician, or holding a specialist position showed significantly better overall performance scores than others (B = 1.881, *p* = 0.012), (B = 1.968, *p* = 0.013), (B = 0.065, *p* = 0.022) respectively. Knowledge scores were also significantly higher among pediatricians (B = 0.064, *p* = 0.003).

Regarding the practice score, those holding the position of medical resident trainee were negatively associated with practice scores (B = 0.047, *p* = 0.032). Years of experience were positively associated with practice scores, which means the experience can improve practice. The practice score was negatively associated with gender, which means that females had better practice scores. The P-value was not significant for all the mentioned variables.

Being a pediatrician or holding a specialist position was associated with a more positive attitude toward COVID-19, reflected in a higher attitude score (B = 0.068, *P*-value 0.002) (B = 0.087, *p* < 0.001), respectively.

## Discussion

This study details Abu Dhabi healthcare providers' knowledge, attitude, and practice that reflects their preparedness for the COVID-19 pandemic very early in its course. The result of this study was similar to the other two studies done among HCWs in the UAE, which showed a similar gap in knowledge and modest attitude. Nevertheless, both were on a smaller number and mainly assessed knowledge and attitude [10, 14]. Reports from other countries revealed similar gaps [15, 16].

Only 9.2% scored more than 30 out of 40, indicating that they have good knowledge. Of the participants, 41.8% had intermediate knowledge scores between 20 and 30 out of 40. These findings suggest gaps in knowledge, practice, and attitudes. This can be explained by the fact that this study was carried out during the early COVID-19 outbreak in the UAE. Moreover, some questions measured multiple aspects simultaneously. The poor overall knowledge score was similar to a study carried out in the United Arab Emirates, which reported a significant knowledge gap between the amount of information available and the depth of knowledge about COVID-19 among HCWs [11].

It is reassuring that most high-risk assessment situations were identified by most of our healthcare providers. Moreover, low-risk assessment exposure was overestimated as high-and moderate-risk exposures.

Specialists had the best scores for knowledge and practice, positive attitude, and years in practice were not related to better knowledge or practice. Medical residents had the worst scores of knowledge and practice, highlighting the possible poor level of residents’ preparedness for medical practice. This necessitates modifying the postgraduate training to include competencies related to infection control early in training. Ayinde et al. [9] showed that similar findings of occupation were significantly associated with knowledge. Worth noting is that pediatricians had significantly higher scores for knowledge, practice, and attitude mean. This better performance could be explained by the higher prevalence of respiratory infections in children and the pediatricians’ accumulating skills in minimizing the risk of infection to themselves. But other factors could play a role that needs more studies.

The deficiency in the knowledge level of healthcare providers concluded in this study and others [11, 14, 16] indicates the great importance of improving the overall quality of information on COVID-19 conveyed not only in HCWs but also in the general population [17].A major contributor to this deficiency in knowledge is that international health agencies lack consistency in the information realized. In a review conducted in March 2020, very early in the pandemic, Saiful Islam et al. reviewed global COVID-19 IPC guidelines by organizations such as the WHO, CDC, and the European Centre for Disease Prevention and Control (ECDC). Guidelines from two high-income countries (Australia and United Kingdom) and one middle-income country (China). They found that recommendations conflict with each other. For example, most guidelines recommend surgical masks for healthcare providers during routine care and N95 respirators for aerosol-generating procedures. However, recommendations regarding the type of face mask varied, and the CDC recommends cloth masks when surgical masks are unavailable [18].

Poor scores reported in this study might be related to the fact that the questionnaire was based on case-based scenarios and did not include many direct questions. Nevertheless, the simulation could be more reflective of the participants' intent. This study utilizing case-based scenarios could be a better design to successfully highlight the critical gaps and associations. There is no study similar to its design that could be identified. Future research is needed to validate this tool by using it as an initial assessment tool of health care providers and then linked to their practice. If valid, such a tool can predict gaps and risky environments for healthcare providers and patients. Educational programs in infection control may benefit from a similar approach with stratification of risk and responses tailored to it based on evidence-based recommendations. Simulated real-life case drills are an opportunity to prepare the healthcare setting for similar infectious disease outbreaks.

## Conclusion

The study-demonstrated gaps in specific aspects of knowledge and practice that need to be focused on in future HCP awareness programs. Interventions are required targeting all HCPs, including physicians, nurses, pharmacists, and technicians, to have good clinical knowledge and practice about COVID-19. Periodic serial assessments similar to this study are well needed to ensure a satisfactory level has been reached in healthcare providers' preparedness for COVID-19 prevention in their workplace.

### Limitations of the study

The participants in the study were mainly from Abu Dhabi City. This limits the generalizability of the study findings to other UAE emirates, although the respondents were from the public and private sectors. Social desirability bias may have occurred because the questionnaire was self-administered. However, the anonymity of the questionnaires was maintained. Another limitation is that a simple linear regression does show associations but not necessarily causality.

## Supplementary Information

**Additional file 1.** "Knowledge, Attitude, and Practices of Healthcare Providers in Abu Dhabi Health Services co. (SEHA) Facilities towards COVID-19 Infection".

## Data Availability

Data are available on request due to restrictions. All relevant data are presented in the paper.

## Abbreviations

COVID-19: Coronavirus Disease-2019
WHO: World health organization
KAPs: Knowledge, attitudes, and practices
OR: Odds ratio
HCW: Healthcare worker
HCPs: Healthcare providers
PPE: Personal protective equipment
SEHA: Abu Dhabi Health Services
CDC: Centers for disease control and prevention
PHQ-9: Patient health questionnaire
GAD-7: General anxiety disorder-7
