# Supplementary material for: COVID-19 knowledge, attitude, and practice of United Arab Emirates heath providers at the start of the pandemic 2020
Source: Arch Public Health. 2023 Feb 13;81:21. doi: 10.1186/s13690-022-01015-w (PMC9924855; doi:10.1186/s13690-022-01015-w)
Supplement: Supplementary file 1 — Additional file 1. "Knowledge, Attitude, and Practices of Healthcare Providers in Abu Dhabi Health Services co. (SEHA) Facilities towards COVID-19 Infection". [file 13690_2022_1015_MOESM1_ESM.docx]

**"Knowledge, Attitude, and Practices of Healthcare Providers in Abu Dhabi Health Services co. (SEHA) Facilities towards COVID-19 Infection"**

*** Age**

*** Gender**

Female

Male

*** Occupation**

Consultant

Specialist

Resident

Nurse

Technician

Pharmacist

*** Specialty**

Family medicine

Internal Medicine

Surgical specialties

Pediatrics

Obstetrics and gynecology

Psychiatry

Other (please specify)

*** Your city where you are practicing?**

*** Your institute?**

*** Your healthcare practice sitting?**

Primary healthcare clinic

In-patient hospital based

Emergency and ICU care

Other (please specify)

*** Years of work experience:**

Read the following case scenarios and answer the related questions:

*** 1. You are working in a primary care clinic. Your patient Ali presents asking for COVID-19 test. Ali’s wife have arrived at the UAE last week. She was febrile and tested positive for COVID-19 infection and was admitted to Alain hospital. Ali is asymptomatic.
A. What is your next action? (Choose all that apply)**

Instruct the patient to wear face mask

Report the suspected case through DOH notification system and fill the required forms

Implement PPE with contact and airborne precaution whenever coming in contact with this patient

Order chest X ray or chest CT imaging

Discharge home, active monitoring for 14 days, while awaiting the results

Send Ali to the nearest ER

I don’t know

*** B. Ali COVID-19 test came negative and he is having high fever, what is your next step (choose one answer only):**

Repeat the test after 24-48 hours

Repeat the test after 14 days

Call back to admit, even if the result is negative

Follow positive case management workflow

I don’t know

*** Ali tested positive at day 12. Nadia, the nurse who triaged Ali in the initial visit was not wearing full PPE and Ali was not wearing a face mask.

C. What is her risk assessment category?**

Low

Medium

High

I don't know

*** 2. Ahmed is 29 years old dentist working in a primary care clinic. The infection control officer contacted him because he did tooth extraction for a case of COVID-19. Total exposure time was 20 minutes.  He was wearing surgical mask and hand gloves. Ahmed now is concerned about his risk of getting COVID-19 infection.
A. According to Ahmed exposure which category he is at?**

Low

Medium

High

I don't know

*** B. What should Ahmed do next? (Choose all that apply)**

Exclude from work for 14 days after last exposure

See his PCP as soon as possible to do COVID-19 test

Take 3 days sick leave until the initial testing result released

Active monitoring for 14 days

Be reassured that until the patient is tested and confirmed positive and symptomatic he continue his life the same

Approach OH clinic for COVID-19 test

I don’t know

*** 3. Mathew is a pharmacist who had a brief conversation with a confirmed COVID-19 patient at the center who was coming to collect medicine from the pharmacy. He recalls that the patient was not wearing mask and was in hurry, while Mathew was not wearing any PPE during that conversation.

A. According to the risk assessment tool, Mathew is in which category:**

Low

Medium

High

I don't know

*** B. Any healthcare provider in the low-risk category should:**

Exclude from work for 14 days after last exposure

Active monitoring for any fever, or respiratory symptom

Continue to work and recommend "self with delegated supervision"

I don’t know

*** 4. Diana is a 45 years old female working in the pediatric ward, you notice that she have dry cough. She have no history of travel or contact with positive COVID-19 case.
A. What is your advice for her?**

Isolate her and send her home

Do COVID-19 PCR

Continue to work no need for anything

I don't know

*** 5. Rashid is a 28 years old medical resident working in the ER, he was exposed to a 60 years old patient diagnosed with COVID-19 pneumonia. He was wearing full PPE.
What you should do for him next? (Choose all that apply)**

Admit to the ward with negative pressure room

Refer to do COVID-19 testing

Continue working with self-delegated supervision

Exclude from work for 14 days after last exposure

I don't know

*** 6. Suha is a general practitioner who was told by the triage nurse that a family is coming to do COVID-19 because their neighbor was found positive and their children and neighbor’s children are playing together.
What should Suha do (choose all that apply):**

Wear PPE before the family enter

Take the family to the isolation room

Advise all the family to do COVID-19 test

Advise the family to self-isolate for 14 days and educate them about the precautions

Report the cases to DOH

Reassure the family and send home with self-monitoring and home quarantine

I don't know

*** 7. Sana is a senior medical student, she is attached to your clinic for her Family medicine rotation, and you will be her supervisor today. She asked you if there is any important consideration she should know. 
A. Which of the following statement you have to tell Sana?  (Choose all that apply)**

All employees are advised to check for any signs of illness and notify their supervisor if they become ill

Minimize interaction and interview time with patients

Ensure to wear the face mask while in the clinic

Avoid being in area like: coffee room, or staff changing room

Don't bring un-necessary stuff to the clinic like: your personal lab top, notes, or text books.

Keep your tools like stethoscope in the clinic, and use disinfectant wipes to clean it frequently

I don't know

*** B. During your duty in the clinic, Do you do all the above?**

Never

Some time

Most of the time

Always

*** 8. Which of following is a mode of COVID-19 virus transmission? (Choose all that apply)**

Respiratory droplets

Direct contact with contaminated surfaces

Oral route

I don't know

*** 9. It is less likely to be transmitted if social distancing is:**

1 meter (3 feets) or more

2 meters (6 feets) or more

I don't know

*** 10. Who of the following considered as a high-risk group? ( Choose all that apply)**

Age>60

Smoker

Diabetic patient

Hypertensive patient

Patient receiving chemotherapy

Patient having Asthma or COPD

Pregnant female

Patient with GERD or peptic ulcer disease

Thalassemia carrier

I don't know

*** 11. When comparing the sensitivity and specificity of COVID-19 testing in detecting the virus, which statement is correct? (Choose all that apply)**

Nasopharyngeal swab is recommended over the oropharyngeal swab

Oropharyngeal swap is recommended over the nasopharyngeal swab

Both are equal

Sensitivity of the NP swab is 70%

*** 12. Wearing a surgical mask is indicated for: (Choose all that apply)**

Suspected COVID-19 infection patient

Only patient with respiratory symptoms like: cough or fever

All community

Only medical staff and care giver in close contact with patients

I don't know

*** Over the past two weeks, how often have you been bothered by the following:**

|  | \| Not at all \| \| --- \| | \| Several Days \| \| --- \| | \| More than half the days \| \| --- \| | \| Nearly every day \| \| --- \| |
| --- | --- | --- | --- | --- | --- | --- | --- | --- |
| \| Feeling nervous, anxious or on edge \| \| --- \| |  |  |  |  |
| \| Not being able to stop or control worrying \| \| --- \| |  |  |  |  |
| \| Worrying too much about different things \| \| --- \| |  |  |  |  |
| \| Trouble relaxing \| \| --- \| |  |  |  |  |
| \| Being so restless that it is hard to sit still \| \| --- \| |  |  |  |  |
| \| Becoming easily annoyed or irritated \| \| --- \| |  |  |  |  |
| \| Feeling afraid as if something awful might happen \| \| --- \| |  |  |  |  |

*** Over the past two weeks, how often have you been bothered by the following:**

|  | Not at all | Several Days | More than half the days | Nearly every day |
| --- | --- | --- | --- | --- |
| \| Little interest or pleasure in doing things \| \| --- \| |  |  |  |  |
| \| Feeling down, depressed or hopeless \| \| --- \| |  |  |  |  |
| \| Trouble falling asleep, staying asleep, or sleeping too much \| \| --- \| |  |  |  |  |
| \| Feeling tired or having little energy \| \| --- \| |  |  |  |  |
| \| Poor appetite or overeating \| \| --- \| |  |  |  |  |
| \| Feeling bad about yourself - or that you’re a failure or have let yourself or your family down \| \| --- \| |  |  |  |  |
| \| Trouble concentrating on things, such as reading the newspaper or watching television \| \| --- \| \|  \| |  |  |  |  |
| Moving or speaking so slowly that other people could have noticed. Or, the opposite - being so fidgety or restless that you have been moving around a lot more than usual |  |  |  |  |
| Thoughts that you would be better off dead or of hurting yourself in some way |  |  |  |  |
